# Decreased metalloproteinase production as a response to mechanical pressure in human cartilage: a mechanism for homeostatic regulation

**DOI:** 10.1186/ar2042

**Published:** 2006-09-14

**Authors:** Jordi Monfort, Natalia Garcia-Giralt, María J López-Armada, Joan C Monllau, Angeles Bonilla, Pere Benito, Francisco J Blanco

**Affiliations:** 1 Unitat de recerca en fisiopatologia òssia i articular- Institut Municipal d'Investigació Mèdica (URFOA-IMIM), Hospital del Mar, Universitat Autònoma de Barcelona, Dr. Aiguader 80, 08003-Barcelona, Spain; 2 Osteoarticular and Aging Research Unit, Rheumatology Division, Biomedical Researcher Center, Complejo Hospitalario Universitario Juan Canalejo, Xubias 84, 15006 – A, Coruña, Spain

## Abstract

Articular cartilage is optimised for bearing mechanical loads. Chondrocytes are the only cells present in mature cartilage and are responsible for the synthesis and integrity of the extracellular matrix. Appropriate joint loads stimulate chondrocytes to maintain healthy cartilage with a concrete protein composition according to loading demands. In contrast, inappropriate loads alter the composition of cartilage, leading to osteoarthritis (OA). Matrix metalloproteinases (MMPs) are involved in degradation of cartilage matrix components and have been implicated in OA, but their role in loading response is unclear. With this study, we aimed to elucidate the role of MMP-1 and MMP-3 in cartilage composition in response to mechanical load and to analyse the differences in aggrecan and type II collagen content in articular cartilage from maximum- and minimum-weight-bearing regions of human healthy and OA hips. In parallel, we analyse the apoptosis of chondrocytes in maximal and minimal load areas. Because human femoral heads are subjected to different loads at defined sites, both areas were obtained from the same hip and subsequently evaluated for differences in aggrecan, type II collagen, MMP-1, and MMP-3 content (enzyme-linked immunosorbent assay) and gene expression (real-time polymerase chain reaction) and for chondrocyte apoptosis (flow cytometry, bcl-2 Western blot, and mitochondrial membrane potential analysis). The results showed that the load reduced the MMP-1 and MMP-3 synthesis (*p *< 0.05) in healthy but not in OA cartilage. No significant differences between pressure areas were found for aggrecan and type II collagen gene expression levels. However, a trend toward significance, in the aggrecan/collagen II ratio, was found for healthy hips (*p *= 0.057) upon comparison of pressure areas (loaded areas > non-loaded areas). Moreover, compared with normal cartilage, OA cartilage showed a 10- to 20-fold lower ratio of aggrecan to type II collagen, suggesting that the balance between the major structural proteins is crucial to the integrity and function of the tissue. Alternatively, no differences in apoptosis levels between loading areas were found – evidence that mechanical load regulates cartilage matrix composition but does not affect chondrocyte viability. The results suggest that MMPs play a key role in regulating the balance of structural proteins of the articular cartilage matrix according to local mechanical demands.

## Introduction

Articular cartilage is a tissue optimised for bearing mechanical loads. Chondrocytes are the only cells present in mature cartilage and they are responsible for the synthesis and integrity of the extracellular matrix (ECM) [1,2]. The matrix of hyaline articular cartilage is composed mainly of proteoglycans (PGs) and type II collagen. The PGs provide elasticity to the tissue, whereas the collagen fibrils form a network that confers tensile strength. Changes in these structural components can affect the mechanical stability of the tissue and chondrocyte survival [3], which consequently may fail to support mechanical loads. The final phase of osteoarthritis (OA) seems to reflect a failure of the reparative process, resulting in degradation of the matrix, cell death, and total loss of cartilage integrity.

Matrix metalloproteinases (MMPs) are involved in the degradation of the components of the cartilage matrix. Among MMPs, collagenase-1 (MMP-1) cleaves a variety of collagens such as collagen I, II, III, VII, and X, and stromelysin-1 (MMP-3) cleaves a variety of ECM components, including certain PGs, collagens, and procollagens [4]. In addition to its proteolytic activity, MMP-3 can activate itself and other MMPs [5] such as MMP-1. MMP-1 and MMP-3 have been implicated in OA [6-9]. Among the earliest changes to cartilage in OA is a loss of PGs, primarily due to proteolytic cleavage of the aggrecan core by MMPs and aggrecanases [10,11]. The breakdown of type II collagen appears at late stages of OA after PG depletion and increases significantly with the severity of the disease [12,13].

Apoptosis, or programmed cell death, differs from necrosis. Apoptosis is involved in the maintenance of homeostasis in adult and embryonic tissue [14]. However, chondrocyte apoptosis has been related to the development of OA [15,16]. Chondrocyte death has a significant role in the development of OA and in the repair of the ECM [17]. A direct relationship between the severity of OA and the frequency of apoptotic chondrocyte death has been observed [18].

The mechanical loading generated during daily activity is a fundamental stimulus for the activity of chondrocytes. Articular cartilage responds to increased mechanical demands by changing the composition of its organic matrix [19-24]. Although mechanical load is a known regulatory factor of cartilage metabolism, the role of proteolytic enzymes in the integrity of matrix maintenance is unclear. Loading effects on cartilage have been widely studied *in vitro *[19-23,25-29]. Although these studies have allowed monitoring of cellular response under closely controlled loading conditions, they have generated inconsistent results due to experimental variation, namely in the tissue evaluated (that is, anatomical location of tissue harvest, species, and age) and test conditions used (for example, loading pressure, time, and frequency and the mechanism used to apply pressure). *In vivo *research, performed primarily with animals, has also led to controversial results due to physiological differences between the species studied [24,30-35]. Furthermore, the results obtained from animals cannot always be extrapolated to humans. Bjelle [36] has analysed the mechanical response of human knees and found an increase in glycosaminoglycan production in load-bearing areas. On the other hand, there are no human *in vivo *studies relating, in the hip joint, the grade of apoptosis with the biomechanical loads.

With this study, we aimed to elucidate the role of MMP-1 and MMP-3 in cartilage composition and to analyse the apoptosis of chondrocytes in response to mechanical load in articular cartilage obtained from maximum- and minimum-weight-bearing regions of human femoral heads. The results suggest that MMPs play a key role in regulating the balance of structural proteins of the articular cartilage matrix according to local mechanical demands. Moreover, no differences in apoptosis levels were found for femur poles, suggesting that mechanical load regulates cartilage matrix composition but does not affect chondrocyte viability.

## Materials and methods

### Obtaining articular cartilage

Human articular cartilage was obtained from hip joints after hip replacement under institutional review. All subjects provided written informed consent before being included in the study. Osteoarthritic specimens were collected from patients with primary symptomatic OA diagnosed by American College of Rheumatology criteria [37], and normal cartilage samples were collected from patients with osteoporotic fracture (OF) with no history of joint disease and with macroscopically normal cartilage. Patients with inflammatory pathology, crystal deposition diseases, osteonecrosis, hip dysplasia or malalignment, or senile ankylosing vertebral hyperostosis (Forestier's disease), as well as patients receiving corticoids or SYSADOA (slow-acting drugs that can modify the symptoms of OA), were excluded. A total of 17 OA and 14 OF femoral heads were used to analyse the ECM proteins (Table 1), and a different joint set of 19 OA and 14 OF femoral heads was employed to carry out experiments focused on apoptosis (Table 2).

### General procedure

The cartilage was dissected from subchondral bone and separated into zones of maximum and minimum mechanical load according to the topographic division of Li and Aspden [38]. Cartilage samples from high loaded areas were named superior pole (SP) and those from low loaded areas were named inferior pole (IP). The laminate tissue was cryopreserved at -80°C until use for protein analysis. The cartilage used for RNA quantification was diced and incubated with RNAlater (Qiagen Inc., Valencia, CA, USA) overnight at 4°C prior to storage at -80°C. Femoral heads lacking sufficient cartilage to perform RNA and protein quantification were discarded. Cartilage was digested to isolate chondrocytes. Recently isolated chondrocytes (non-culture chondrocytes) were used to quantify apoptosis and mitochondrial depolarisation.

### Extraction and quantification of cartilage matrix proteins

The procedure was performed according to a previous study [39] with some modifications. Briefly, cartilage samples were suspended in an appropriate volume of cold extraction buffer (0.05 M Tris-HCl [pH 7.5], 0.1% CHAPS (3- [(3-cholamidopropyl)dimethylammonio]propanesulfonate), and Complete EDTA (ethylenediaminetetraacetic acid)-free protease inhibitor cocktail [Roche Diagnostics, Basel, Switzerland]) to obtain 10% (wt/vol) total cartilage homogenate. Samples were homogenised using a T8 Ultra-Turrax homogeniser (IKA Works, Inc., Wilmington, NC, USA). A 200-μl volume of homogenate was then mixed with 100 μl of 8 M guanidine hydrochloride, and 500 μl of 0.05 M Tris-HCl (pH 7.5) was added to the mixture. The solution was incubated overnight at 4°C with constant stirring before centrifugation at 10,000 *g *for 5 minutes at 4°C. The supernatant, containing the soluble fraction of cartilage matrix (PGs and MMPs), was carefully removed, aliquotted into separate tubes, and stored at -80°C until use. The pellet, which contained the collagen fibers, was washed extensively with cold distilled water and dissolved for native type II collagen detection according to the manufacturer's protocol (Chondrex, Inc., Redmond, WA, USA, distributed by MD Biosciences, Zürich, Switzerland). The supernatant aliquots were used to determine the concentrations of aggrecan (PG EASIA; BioSource Europe S.A., now part of Invitrogen Corporation, Carlsbad, CA, USA), MMP-1 and MMP-3 (Biotrak ELISA System; Amersham Biosciences, now part of GE Healthcare, Little Chalfont, Buckinghamshire, UK) by enzyme-linked immunosorbent assay (ELISA) according to the manufacturer instructions, and total soluble protein via Bradford's method [40]. This last value was used to normalise the respective ELISA data for comparing the samples.

### Total RNA isolation and gene expression quantification of cartilage matrix proteins

Cartilage samples (50 mg) were suspended in 1 ml of Tri Reagent (Molecular Research Center, Inc., Cincinnati, OH, USA) and homogenised using a T8 Ultra-Turrax homogeniser (IKA Works, Inc.) and, finally, the RNA was extracted according to Tri Reagent manufacturer instructions. Total RNA was quantified at 260 nm, and 150 ng was used to synthesise the DNA complementary strain according to the protocol of TaqMan^® ^Reverse Transcription Reagents (Applied Biosystems, Foster City, CA, USA). The product was diluted by half with RNAse-free pure water, and 1 μl of the resultant solution was used to determine gene expression (aggrecan [AGC1], (type II collagen [COL2A1], and MMP-1 and MMP-3) using quantitative real-time polymerase chain reaction (PCR). Briefly, real-time PCR was conducted in a volume of 20 μl containing gene-specific Assay on Demand primers and TaqMan-MGB probe and 10 μl TaqMan Universal PCR MasterMix 2X (Applied Biosystems) in the following sequence: 2 minutes at 50°C, followed by 50 cycles at 95°C for 15 seconds, and then at 60°C for 60 seconds in 384-well plates with the ABI PRISM 7900 HT Detection System (Applied Biosystems). Results were analysed using the SDS software version 2.1 (Applied Biosystems), and expression levels were calculated versus 18S expression (relative expression) using arbitrary units. All real-time PCRs for each sample were performed in triplicate. Real-time PCR for 18S was carried out under the same conditions, using an 18S endogenous control Assay on Demand (Applied Biosystems).

### Chondrocyte viability analysis

The cartilage surfaces were rinsed with saline and sliced full thickness, excluding the mineralised cartilage and the subchondral bone. To isolate the cells, the cartilage surfaces were rinsed with saline and the tissue was incubated at 37°C with trypsin for 10 minutes. After the trypsin solution was removed, the cartilage slices were treated with type IV collagenase (2 mg/ml) (Sigma-Aldrich, St. Louis, MO, USA) for 12 to 16 hours. Human chondrocytes were recovered, cell viability was assessed by tryplan blue dye exclusion, and chondrocytes were employed to quantify apoptosis and mitochondrial depolarisation.

### DNA labeling technique for flow cytometric analysis

Cells were fixed in 70% ethanol at 4°C for 60 minutes, washed with water, incubated with RNAse (50 μg/ml) and propidium iodide (PI) (100 μg/ml) for 15 minutes at room temperature in the dark, and then stored at 4°C. PI fluorescence of nuclei was measured by flow cytometry (FAC) on a FACScan (Becton Dickinson, Mountain View, CA, USA) using a 560-nm dichromatic mirror and a 600-nm band-pass filter. Data are expressed as percentage apoptotic (hypodiploid) nuclei in total cell population.

### Determination of mitochondrial membrane potential

The fluorescent probe JC-1 (5, 5', 6, 6'-tetrachloro-1, 1', 3, 3'-tetraethylbenzimidazole carbocyanide iodide) was used to measure the mitochondrial membrane potential (Δψm) of chondrocytes. JC-1 exists as a monomer at low values of Δψm (green fluorescence) but forms aggregates at high Δψm (red fluorescence). Thus, for mitochondria with normal Δψm, JC-1 forms aggregates (red fluorescence), whereas with de-energised or depolarised Δψm, JC-1 remains a monomer (green fluorescence).

Chondrocytes (5 × 10^5^) were washed in phosphate-buffered saline (PBS) (pH 7.4) and incubated with 10 μg/ml JC-1 at 37°C for 15 minutes. Cells were pelleted at 200 *g *for 5 minutes, washed in PBS, and then analysed by FAC using a FACScan and Cell-Quest software (Becton Dickinson). The analyser threshold was adjusted on the forward scatter channel to exclude the majority of subcellular debris. Photomultiplier settings were adjusted to detect JC-1 monomer fluorescence signals on the FL1 detector (green fluorescence, centered at approximately 390 nm) and JC-1 aggregate fluorescence signals on the FL2 detector (red fluorescence, centered at approximately 340 nm). The data were analysed with Paint-a-Gate Pro software (Becton Dickinson). Mean fluorescence intensity values for FL1 and FL2, expressed as relative linear fluorescence channels (arbitrary units scaled from channels 0 to 10,000), were obtained for all experiments. In each experiment, at least 20,000 events were analysed. The relative aggregate/monomer (red/green) fluorescence intensity values and percentage depolarisation were used for data presentation.

### Western blot

Cells were washed in ice-cold PBS (pH 7.5) and lysed in 0.2 M Tris-HCl (pH 6.8) containing 2% SDS, 20% glycerol, 1 μg/ml cocktail inhibitor (Sigma-Aldrich), and 1 mM PMSF (phenyl methyl sulfonyl fluoride) (Sigma-Aldrich). Whole-cell lysates were boiled for 5 minutes, and protein concentrations were determined using a BCA (bicinchoninic acid) reagent assay (Pierce Biotechnology, Inc., Rockford, IL, USA). The protein extracts (30 μg) were resolved on 12.5% SDS-polyacrylamide gels and transferred to polyvinylidene difluoride membranes (Immobilon P; Millipore, Billerica, MA, USA). Membranes were first blocked in Tris buffered saline (pH 7.4) containing 0.1% Tween-20 and 5% non-fat dried milk for 60 minutes at room temperature and then incubated overnight with anti-bcl-2 (mouse anti-human bcl-2; R&D Systems Europe Ltd, Abingdon, Oxfordshire, UK) at 4°C. After washing, the membranes were incubated with peroxidase-conjugated secondary antibodies and developed using an ECL chemiluminescence kit (GE Healthcare). To ensure that equal amounts of total proteins were charged, we also hybridised each membrane with anti-tubuline (Sigma-Aldrich).

### Data analysis

Data was analysed with SSPS 10.0 software (SPSS Inc., Chicago, IL, USA). The ratio of aggrecan to type II collagen was calculated from ELISA data. Both sets of data were obtained from the same protein extraction tube: the PG was located in the supernatant, whereas the type II collagen was located in the pellet. MMP-1 and MMP-3 data obtained from ELISAs were normalised using data from total soluble protein quantification after PG extraction. Thus, the ELISA values were not representative of the protein concentration in the tissue. The results were expressed as a percentage of total protein content. Real-time PCR results were normalised using the endogenous control 18S and the same sample was used for relative quantification. Apoptosis results were expressed as mean ± standard deviation. Individual donors were studied in triplicate; cells from different donors were not pooled in any experiment.

To examine the statistical significance of differences between cartilage areas (that is, SP versus IP), pair-wise comparisons between poles from the same sample were assessed using the Wilcoxon paired-sample test. The test was used to reduce the variance due to the high inter-individual variability. Differences between OA and OF cartilages were evaluated using the Mann-Whitney *U *test. *P *values less than 0.05 were considered significant.

## Results

### Effect of load on matrix cartilage

**Figure 1:**
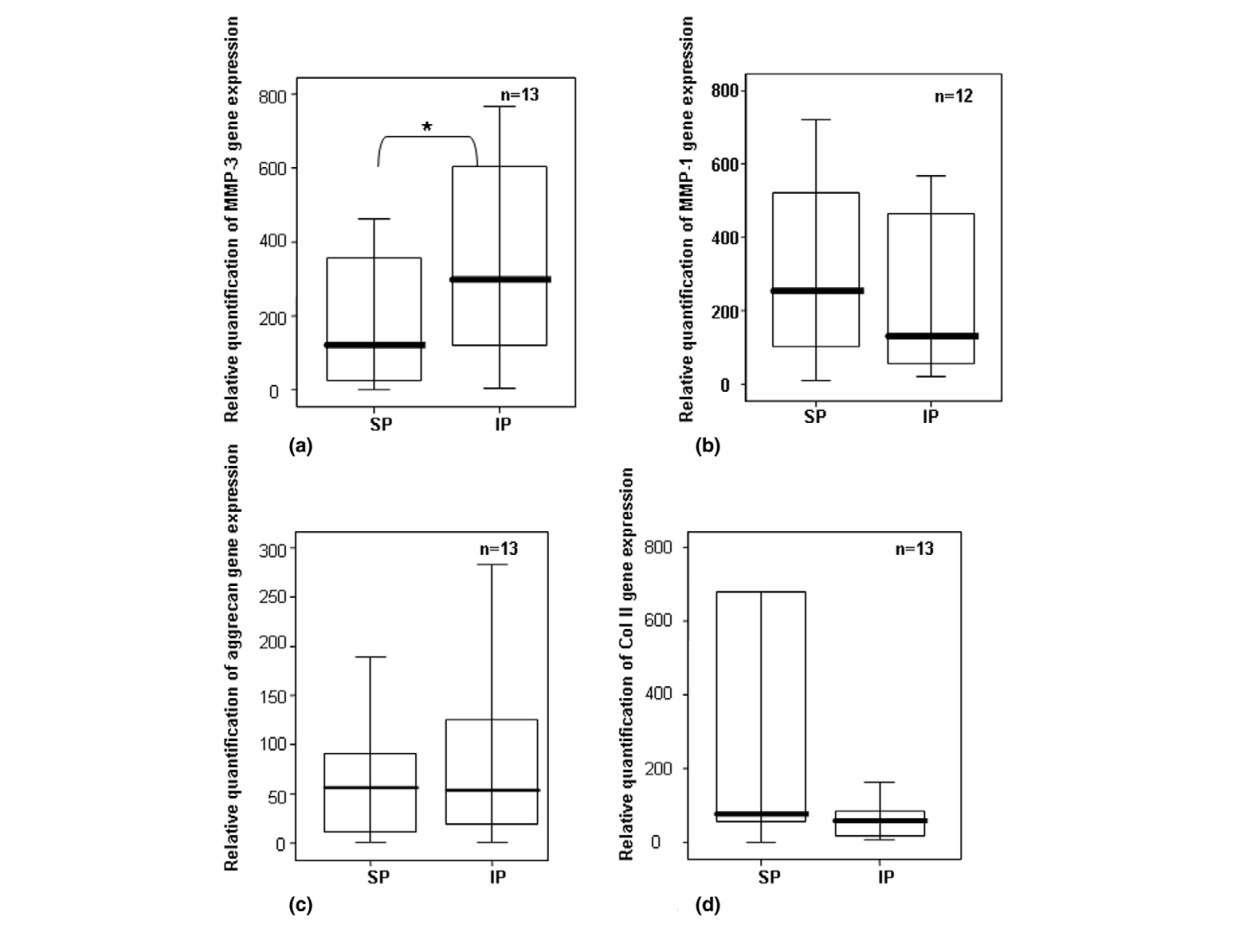
Gene expression of MMPs, aggrecan, and type II collagen. Quantification of gene expression of **(a) **MMP-3, **(b) **MMP-1, **(c) **aggrecan, and **(d) **type II collagen in chondrocytes from normal human femoral heads using real-time polymerase chain reaction. 18S rRNA was used as endogenous control, and the results are relative to a certain sample pertaining to the experiment. Separated maximum (SP) and minimum (IP) mechanical load areas were obtained from each femoral head. The horizontal bar shows the median, the box is the interquartile range, and the vertical lines show the atypical values. The Wilcoxon signed rank test was used to compare areas within the same joint. *Significant differences between poles, in MMP-3 values, were found, *p *< 0.05. No significant differences were found in MMP-1, aggrecan, and type II collagen gene expression. IP, inferior pole; MMP, matrix metalloproteinase; n, number of femoral heads used in the experiment; SP, superior pole.

**Figure 2:**
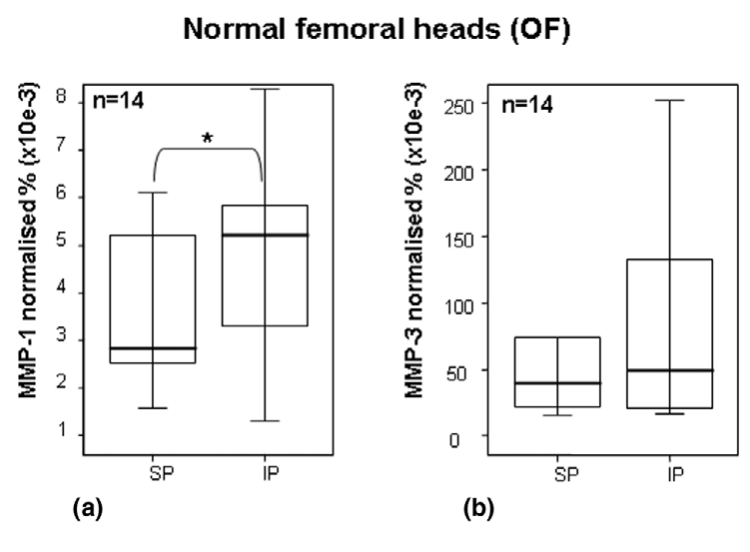
Quantification of MMP-1 and MMP-3 by ELISA in OF cartilage. Quantification of **(a) **MMP-1 and **(b) **MMP-3 in articular cartilage from normal human femoral heads using ELISA. Values were normalised to total soluble protein, which was obtained after proteoglycan extraction and was quantified by Bradford method. Separated maximum (SP) and minimum (IP) mechanical load areas were obtained from each femoral head. The horizontal bar indicates the median, the box is the interquartile range, and the vertical lines indicate the atypical values. Median values were expressed as percentages. The Wilcoxon signed rank test was used to compare areas within the same joint. *Significant differences in MMP-1 values between areas were found (*p *< 0.05). No significant differences were found in MMP-3 values. ELISA, enzyme-linked immunosorbent assay; IP, inferior pole; MMP, matrix metalloproteinase; n, number of femoral heads used in the experiment; OF, osteoporotic fracture; SP, superior pole.

**Figure 3:**
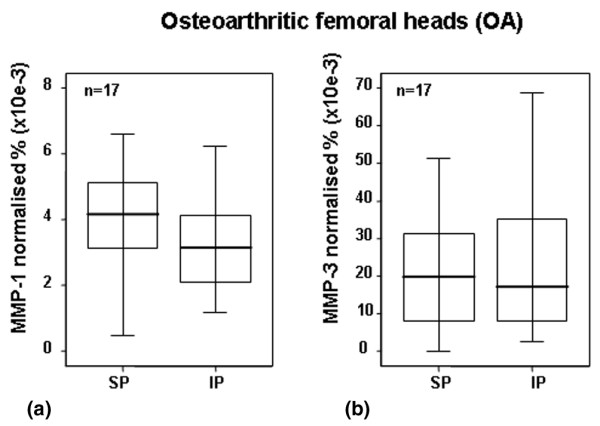
Quantification of MMP-1 (a) and MMP-3 (b) by ELISA in OA cartilage. Quantification of MMP-1 and MMP-3 in articular cartilage from OA human femoral heads using ELISA. Values were normalised to total soluble protein, and medians were expressed as percentages. Separated maximum (SP) and minimum (IP) mechanical load areas were obtained from each femoral head. The horizontal bar indicates the median, the box is the interquartile range, and the vertical lines indicate the atypical values. The Wilcoxon signed rank test was used to compare areas within the same joint. No significant differences between areas were found. ELISA, enzyme-linked immunosorbent assay; IP, inferior pole; MMP, matrix metalloproteinase; n, number of femoral heads used in the experiment; OA, osteoarthritis; SP, superior pole.

**Figure 4:**
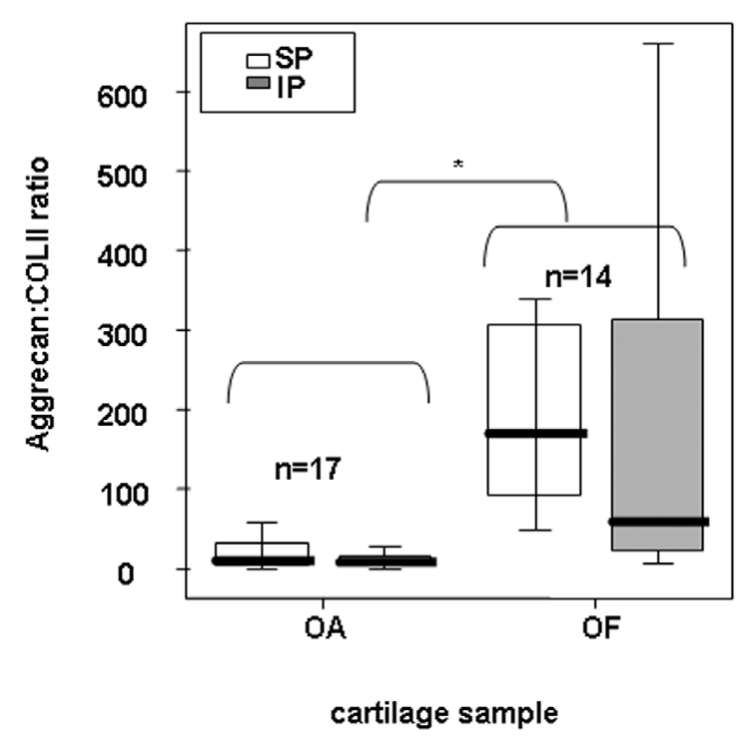
Analysis of aggrecan and type II collagen in OF and OA cartilage. Ratio of aggrecan to type II collagen in the cartilage matrix of OA and OF femoral heads and comparison between areas (SP and IP). Aggrecan and type II collagen were quantified using ELISA after cartilage proteoglycan extraction. Aggrecan was assessed from supernatant soluble fraction, and collagen was assessed from pellet fraction of the same tube. The horizontal bar indicates the median, the box is the interquartile range, and the vertical lines indicate the atypical values. *Significant differences between OA and OF heads were found (*p *< 0.05) using the Mann-Whitney *U *test. These differences were found for both poles. No significant differences between weight-bearing areas were found, but a trend was found between poles for OF hips (*p *= 0.057) using the Wilcoxon signed rank test. ELISA, enzyme-linked immunosorbent assay; IP, inferior pole; n, number of femoral heads used in the experiment for each cartilage condition (osteoarthritis or osteoporotic fracture); OA, osteoarthritis; OF, osteoporotic fracture; SP, superior pole.

**Figure 5:**
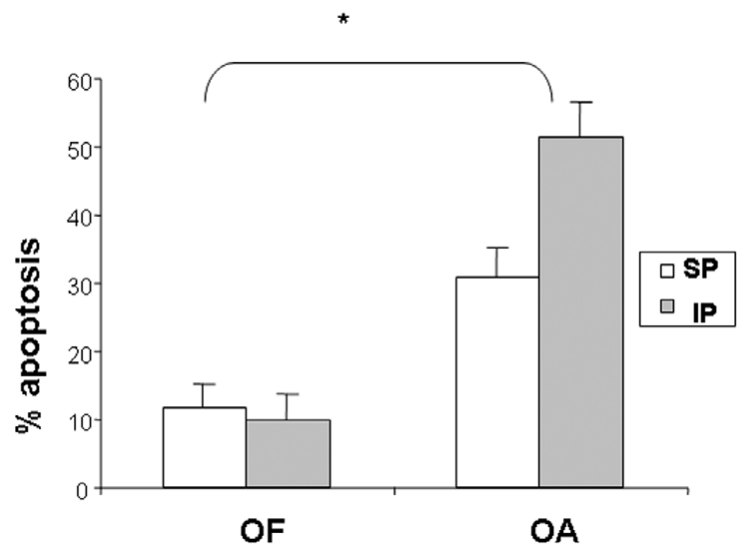
Quantification of apoptosis in OF and OA cartilage. Quantification of apoptosis. Percentage of apoptotic cells measured by flow cytometry of OA (*n *= 6) or OF (*n *= 3) femoral heads and comparison between areas (SP and IP). No differences were found between areas for either set of cartilage conditions. *Significant differences (*p *< 0.05) were found between OA and OF cartilage. IP, inferior pole; OA, osteoarthritis; OF, osteoporotic fracture; SP, superior pole.

**Figure 6:**
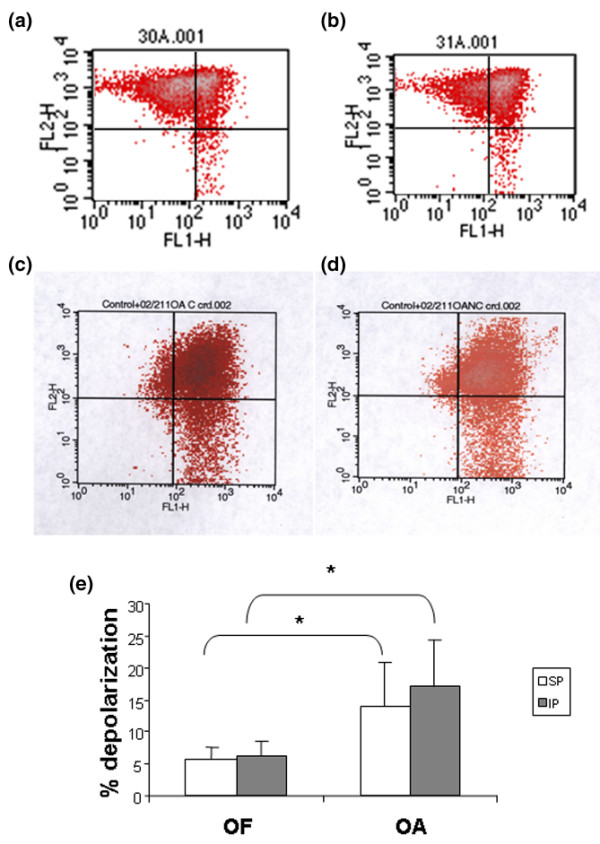
Mitochondrial depolarisation in OF and OA chondrocytes. Percentage depolarisation of OA or OF femoral heads and comparison between areas (SP and IP). **(a, b) **OF cartilage. **(a) **and **(b) **show results of chondrocytes from SP and IP zones, respectively. **(c, d) **OA cartilage. **(c) **and **(d) **show results of chondrocytes from SP and IP zones, respectively. **(e) **Quantification of mitochondrial depolarization. No differences were found between areas for either set of cartilage conditions. *Significant differences were found between OA and OF cartilage. IP, inferior pole; OA, osteoarthritis; OF, osteoporotic fracture; SP, superior pole.

**Figure 7:**
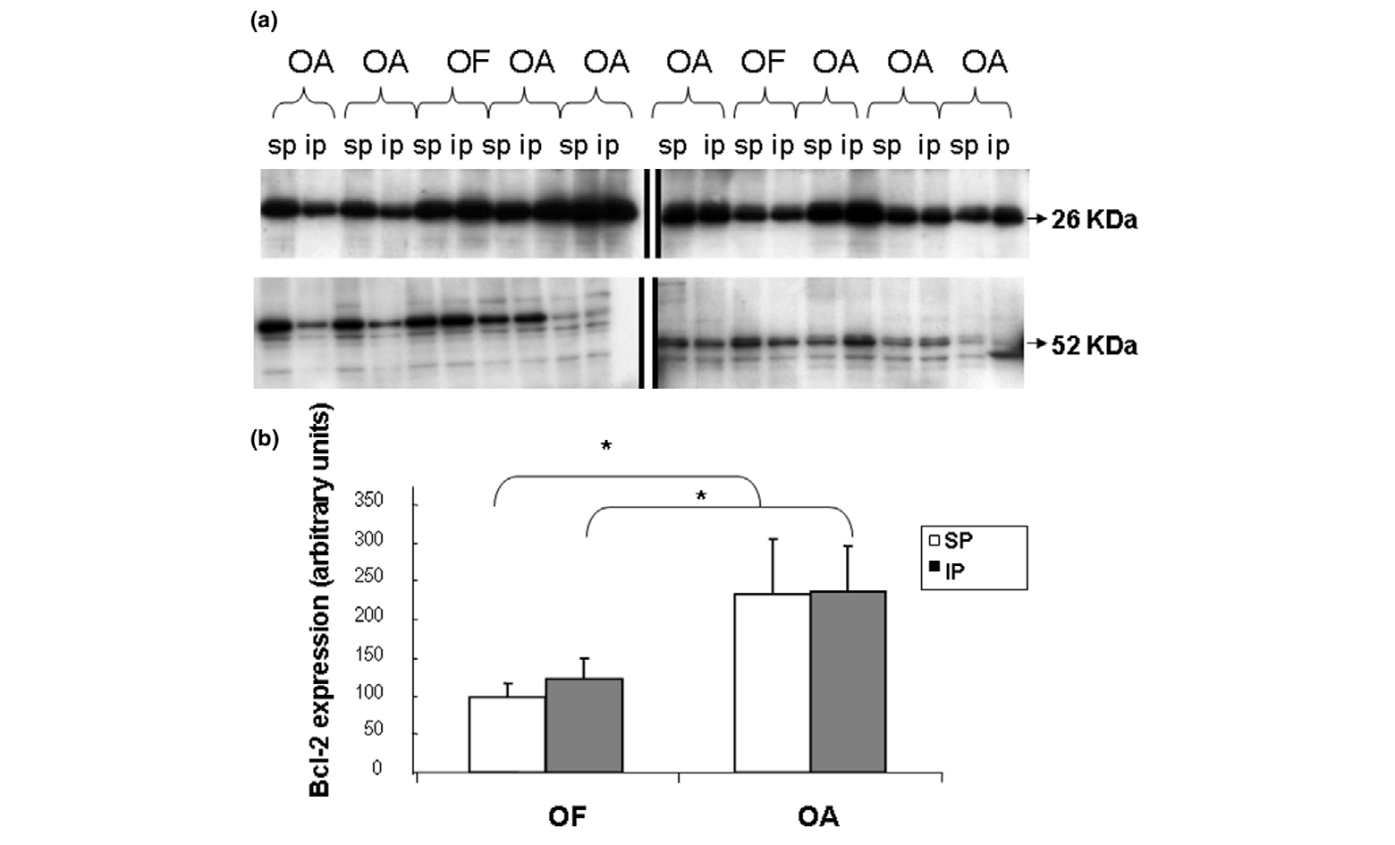
Analysis of bcl-2 in OA and OF chondrocytes. Western blot of bcl-2 in OA or normal (OF) chondrocytes and comparison between areas (SP and IP). **(a) **Aliquots of total cell lysates were subjected to SDS-PAGE; immunoblotting was performed using anti-blc-2 antibody as described in Materials and methods. Molecular size markers are shown on the left (26 kDa = bcl2; 52 kDa = Tubulin). Data are representative of four separate experiments. **(b) **Percentage of basal protein expressed as arbitrary densitometric units. Levels of bcl-2 protein were significantly higher in OA cartilage than in normal cartilage. IP, inferior pole; OA, osteoarthritis; OF, osteoporotic fracture; SP, superior pole.

Because data from ELISA and from real-time PCR did not follow a normal distribution, according to the K-S test (*p *< 0.05), and the variances were not homogeneous, according to the Levene test (*p *< 0.05), non-parametric tests were used. The Wilcoxon signed rank test was used to compare related samples obtained from the same joint (SP versus IP), whereas the Mann-Whitney *U *test was used to analyse independent samples of observations (OF or OA femoral heads). Both statistic tests are recommended for small samples. These tests analyse the median difference (Figures 1, 2, 3, 4, 5, 6, 7) in paired data (OA versus OF or SP versus IP).

The first set of experiments was centred to analyse variations in mRNA levels between poles via real-time PCR. The results, in healthy cartilage, showed that the load reduced the mRNA levels of MMP-3 (Figure 1a), whereas no differences were found in MMP-1 gene expression (Figure 1b). No significant differences between pressure areas were found in aggrecan (Figure 1c) or type II collagen gene expression levels (Figure 1d). However, COL2A1 showed increased mRNA levels in the weight-bearing areas (mean ± standard error [SE]: SP = 2378.55 ± 1562.23; IP = 83.3 ± 31.36). When areas from OA cartilage were analysed, no differences between poles were found for MMP-1 or MMP-3 (data not shown).

The next set of experiments was carried out to analyse the effect of loading on the levels of MMP-1 and MMP-3 protein synthesis. Because the ELISA kit used to detect MMP screens the total MMP content (that is, free MMP, proMMP, and MMP/TIMP [tissue inhibitor metalloproteinase] complexes), we could not discriminate between active and inactive forms of MMP. MMP-1 quantification revealed that the load reduced the protein level (*p *< 0.05) (Figure 2a). In the case of MMP-3, no significant differences were found, although a subtle increase in MMP-3 protein levels was observed at the inferior (non-weight-bearing) pole (Figure 2b). The MMP-3 protein results were in concert with the results observed for mRNA assessment (Figure 1a). When areas from OA cartilage were analysed, no differences between poles were found for MMP-1 or MMP-3 (Figures 3a and 3b, respectively). In all cases, the quantity of MMP-1 in the matrix was inferior to that of MMP-3 (Figures 2 and 3).

To evaluate the balance between the major matrix proteins in normal and in pathologic cartilage, the ratio of aggrecan to type II collagen was determined by ELISA. This ratio allows normalisation of data and elimination of variability due to cartilage quality, cartilage wet weight, and experimental variation. Significant differences (*p *< 0.001) were found when OA and OF cartilages were compared (Figure 4). These differences were found for both poles. Compared with normal cartilage, OA cartilage showed 10- to 20-fold less aggrecan with respect to type II collagen. This variation reflected an imbalance of the proteins in human OA cartilage. No significant differences between weight-bearing areas were found for OF or OA cartilage (Figure 4). However, a trend was found for OF hips (*p *= 0.057), using the signal test: IP was observed at a lower ratio than SP (approximately equal to three folds), and the proportion was maintained independently of cartilage condition.

### Effect of load on chondrocytes

Normal cells had diploid DNA, whereas apoptotic cells contained low-molecular weight DNA. FAC results obtained from six samples of OA chondrocytes showed a mean percentage of apoptotic cells ± SE of 30.92% ± 4.12% in maximum-weight-bearing regions and 51.4% ± 5.23% in minimum-weight-bearing regions. FAC results from three samples of OF chondrocytes showed a mean percentage of apoptotic cells ± SE of 11.7% ± 3.5% in SP and 10.1% ± 3.9% in IP (Figure 5). No differences were found between areas under either set of cartilage conditions. However, significant differences were found between OA and OF cartilage (*p *< 0.05).

Because mitochondria play an important role in programmed cell death, we analysed Δψm values for the loading and non-loading zones of both chondrocyte populations. The same ratio of red/green fluorescence was observed for both groups (1.2 ± 0.3 versus 1.3 ± 0.8) of OA chondrocytes. Moreover, no difference in the percentage of chondrocyte depolarisation was found (load: 13.85 ± 6.92 versus no load: 16.92 ± 7.47). In OF chondrocytes, the ratios of red/green fluorescence were 3.3 ± 1 (load) and 3.1 ± 0.9 (no load), and the percentages of depolarisation were 5.7 ± 1.9 and 6.3 ± 2.1, respectively (Figure 6). No significant differences between the areas were found. However, significant differences (*p *< 0.05) were found between OA and OF chondrocytes. Lastly, synthesis of bcl-2 protein was higher in OA chondrocytes than in normal cells. However, a similar synthesis of bcl-2 was found in loading and non-loading zones (Figure 7a,b).

## Discussion

Under physiological loading conditions, the cartilage matrix suffers compressive, tensional, and shear stress. Appropriate joint loads maintain healthy cartilage with a specific protein composition according to loading demands [32,35]. In contrast, inappropriate loads alter the compositional properties of cartilage, leading to OA [22]. OA is the most common joint disease in humans and is characterised by a progressive loss of articular cartilage in joints. In OA, there is a disruption of the delicate balance between degradation and synthesis of the cartilage ECM which is maintained by chondrocytes. Although the loading effect on OA physiopathology is well known, the mechanisms by which loads affect matrix composition and cell death are unclear. This study was designed to clarify the *in vivo *behaviour of human articular cartilage from femoral heads in response to load. Given that human femoral heads are subjected to different loads *in vivo *at defined sites (the SP is the most highly loaded, whereas the IP is the least loaded [38]), we decided to obtain both areas from the same hip and subsequently to evaluate them for differences in gene expression, protein content, and apoptosis. The use of human samples, as opposed to animal samples, provides information that is more relevant to real human articular cartilage and its physiopathology. On the other hand, studies with human samples can also suffer from wide variability caused by uncontrolled environmental factors. In the present study, samples of both poles were collected from the same hip to minimise inter-individual variability during data comparison.

Several *in vitro *and *in vivo *studies have supported the view that load response is governed by proteases [30,41,42]. Sun *et al*. [42] examined the effects of loading on fibroblast-like synoviocyte cells, focusing on the expression and activity of MMP-1 and MMP-13. The results showed that the cyclic strain reduced the mRNA and protein levels of MMP-1 and MMP-13. Moreover, decreased MMP-2 levels were found in human OA chondrocytes *in vitro *under intermittent hydrostatic pressure [43]. Furthermore, proteinase inhibition has been observed *in vivo *in animal models when cyclic mechanical loads were used [30]. Our results using human cartilage *in vivo *demonstrated that load reduced the protein and mRNA expressions of MMP-1 and MMP-3, respectively (Figures 1 and 2). This finding is consistent with the aforementioned studies. Moreover, MMP-1 levels were much lower than those of MMP-3, confirming the results of the previous report [44]. This work shows the important role of MMPs in loading response *in vivo*. On the other hand, no differences were found between pressure areas in OA cartilage when MMPs were quantified (data not shown). This result suggested that OA cartilage might suffer from a loss of regulation of MMP synthesis, for which we have conceived three hypotheses. In the first scenario, OA cartilage has lower cellularity and consequently a lower response capacity. Another possibility is that OA chondrocytes may be less sensitive to loading response. Lastly, other inflammatory factors may act on chondrocytes mainly as stimuli hiding the load effect. This loss of regulation could be involved in the development of OA.

The normal function of articular cartilage relies on the structural integrity and biochemical composition of the ECM. Aggrecan and type II collagen, the two major structural matrix macromolecules, are critical components of ECM which determine the mechanical properties of the tissue. Given that both content and organisation of these components appear to be related to local functional requirements, the balance between aggrecan and type II collagen could be a critical parameter for matrix integrity. Therefore, articular cartilage areas with different loading demands require different structural protein concentrations. We propose that successful cartilage function depends on how joint loads influence proteinase expression, which could modify the balance between aggrecan and type II collagen.

Different protein compositions were observed as a function of loading demand (Figure 4). The weigh-bearing zones had a higher ratio of aggrecan/type II collagen than did no-loading areas. These results indicated that articular cartilage can change the composition of its organic matrix in response to increased mechanical demands. Others studies agree with these results, showing that higher loads increase the concentration of PGs in articular cartilage [22,34,45]. Elevated amounts of PGs can be expected to amplify tissue elasticity through osmotic effects. Load could increase the concentration of PGs in articular cartilage as a result of increased synthesis and/or reduced degradation. Comparison of the hip poles for gene expression levels revealed lower expression levels of MMP-3 in the SP but no difference in aggrecan gene expression (Figures 1 and 2). If it were the case that gene expression corresponded to real protein synthesis, our results would indicate that differences in aggrecan levels between poles could be a consequence of decreased degradation. However, we cannot discard other post-transcriptional processes in the regulation of protein content.

Load also affects the content of type II collagen. The gene expression results did not show significant differences between poles, but an evident trend was noticed when medians and means were compared (Figure 1d). We hypothesise that the high degree of variability among individuals made it difficult to find significant differences in collagen synthesis. Several *in vitro *studies have shown that mechanical compression enhances the expression of type II collagen [19,22]. However, our results demonstrated that the concentration of type II collagen is regulated by a combination of increased gene expression and reduced degradation by MMP-1 (Figures 1d and 2a). We interpret that the cartilage attempts to repair the effect of the pressure and shear over areas submitted to higher loads. Therefore, pressure could be viewed as a stimulus for ECM protection and maintenance.

OA cartilage often exhibits a decompensate synthesis of the components. OA begins through depletion of PGs and fibrillation of the superficial collagen network at the cartilage surface [45,46]. The breakdown of type II collagen follows the degradation of PGs and is severe at late stages of OA [12,13]. A decrease in the concentration of superficial PG, as well as separation and disorganisation of the superficial collagen fibrils, occurred before deterioration of the cartilage [45]. Our results demonstrated an altered ratio of PG to type II collagen with a drastic depletion of aggrecan in both hip poles. OA cartilage showed lower levels of aggrecan with respect to collagen because the variation in PG content was the first detectable abnormality in the pathogenesis. Accordingly, we detected PG depletion before loss of type II collagen. Alternatively, PG loss may occur prior to loss of collagen, which has been shown to be retained in the fibril after denaturation and cleavage and is therefore not released [12]. However, we cannot know whether the imbalance of PG and type II collagen is a consequence or cause of OA.

Cell death by injurious mechanical load has been observed in both *in vivo *and *in vitro *studies, although some of these studies have reported that apoptosis did not appear to be the cause [2,47]. However, injurious mechanical loading has also been observed to significantly increase the number of apoptotic cells [48,49]. A proportionately similar reduction in the cellularity of the SP and IP in normal femoral heads with age has likewise been described [50]. This reduction seems to be independent of local ambient factors such as biomechanical load. However, no study has been performed to assess the daily loading effect on chondrocyte viability in healthy and OA human cartilage *in vivo*. Therefore, our intention was to elucidate whether the different load-bearing areas had different degrees of cellular apoptosis *in vivo*. The study was performed using three complementary approaches, including the nucleus and mitochondria processes: detection of low-molecular weight DNA, determination of mitochondrial membrane potential, and quantification of the synthesis of bcl-2, an inner mitochondrial membrane protein that blocks programmed cell death.

In human adult normal articular cartilage, cell loss increases with age [50,51] and is greater in OA human cartilage than in normal cartilage [51,52]. Several groups of investigators have shown a relationship between apoptosis and the development of OA cartilage [15,16,18,49,53], reporting that the percentage of apoptotic cells is greater in OA than in normal cartilage and that the percentages in OA cartilage vary with the method used, ranging from an average of 51% [15] to 1.4% [18]. The results obtained in our study corroborate those previously reported in fleshly isolated chondrocytes. The percentage of apoptotic cells in OA and normal cartilage was approximately 44% and 10%, respectively. A hypothesis to explain the high percentage of chondrocyte apoptosis in both normal and OA cells is that the enzymatic digestion process induces or accelerates apoptosis in chondrocytes. It has been reported that collagenase is a pro-apoptotic factor [54,55]. Interestingly, we have not observed differences in the percentages of apoptotic cells between maximum- and minimum-weight-bearing regions in OA or in normal hips, suggesting that load does not influence chondrocyte apoptosis. Curiously, in OA cartilage, the percentage of apoptosis in SP was numerically (but not significantly) higher than in IP. Furthermore, mitochondrial depolarisation showed that OA cartilage has higher levels at both poles than normal cartilage, as was reported [56]. However, Bcl-2 levels were higher in OA cartilage than normal cartilage, confirming the result reported. Finally, we did not find differences in any parameter analysed between both poles in normal cartilage, suggesting that normal loads are not involved in cell-programmed death.

## Conclusion

These data suggest that the synthesis of MMPs plays a key role in the response of human femoral head articular cartilage to mechanical loading. The results show that major load reduced the mRNA and protein levels of MMP-1 and MMP-3. However, a similar role for MMPs was not observed for OA cartilage. Furthermore, the diverse ratios of aggrecan to type II collagen found in the matrix cartilage areas according to load-bearing capacity suggest that the balance between the major structural proteins seems to be crucial to the integrity and function of the tissue. This balance is lost in OA cartilage at both hip poles, and this loss may cause the tissue destabilisation that characterises the pathogenesis of the disease. Our results have not shown a direct relationship between the percentage of apoptotic chondrocytes and the areas of maximal and minimal load of the coxofemoral joint.

## Abbreviations

ECM = extracellular matrix; ELISA = enzyme-linked immunosorbent assay; FAC = flow cytometry; IP = inferior pole; MMP = matrix metalloproteinase; OA = osteoarthritis; OF = osteoporotic fracture; PBS = phosphate-buffered saline; PCR = polymerase chain reaction; PG = proteoglycan; PI = propidium iodide; SE = standard error; SP = superior pole.

## Competing interests

The authors declare that they have no competing interests.

## Authors' contributions

JM was involved in the conception and design of the study, helped to draft the manuscript, and gave final approval of the version to be published. PB was involved in drafting the manuscript and revised it critically for important intellectual content. NGG carried out the experimental procedures of the cartilage matrix part, performed the statistical analysis, and helped to draft the manuscript. JCM conducted the hip replacement, collected the samples, and checked clinical histories for the inclusion and exclusion criteria. MJLA carried out the Western blot experiments. AB carried out the studies centred in mitochondrial despolarisation and quantification of apoptosis by cytometry. FJB conceived of the study, participated in its design and coordination, and helped to draft the manuscript. All authors read and approved the final manuscript.

## Figures and Tables

**Table 1:** Demographic data of cartilage donors

	Osteoporotic fracture		Osteoarthritis	
	
	*n*	Mean age ± SD (years)	*n*	Mean age ± SD (years)
Women	11	77.8 ± 2.49	7	69.6 ± 8.87
Men	3	84 ± 2.31	10	64.6 ± 8.6

Samples used for protein matrix analysis. Gender and mean age ± SD of patients with osteoarthritis and healthy subjects included in this study. SD, standard deviation.

**Table 2:** Demographic data of cartilage donors

	Osteoporotic fracture		Osteoarthritis	
	
	*n*	Mean age ± SD (years)	*n*	Mean age ± SD (years)
Women	13	78.1 ± 3	15	68.8 ± 8
Men	1	81	4	73.5 ± 6

Samples used for apoptotic studies. Gender and mean age ± SD of patients with osteoarthritis and healthy subjects included in this study. SD, standard deviation.
